# Repository corticotropin injection attenuates collagen-induced arthritic joint structural damage and has enhanced effects in combination with etanercept

**DOI:** 10.1186/s12891-020-03609-3

**Published:** 2020-08-31

**Authors:** Dima A. Decker, Paul Higgins, Kyle Hayes, Chris Bollinger, Patrice Becker, Dale Wright

**Affiliations:** 1 Former employee of Mallinckrodt Pharmaceuticals, Bedminster, NJ USA; 2 Mallinckrodt Pharmaceuticals, 675 McDonnell Blvd, Bedminster, NJ 63042 USA

**Keywords:** Rheumatoid arthritis, Melanocortin receptor agonist, Repository corticotropin injection, Bone loss, Etanercept, Osteoclast

## Abstract

**Background:**

Melanocortin receptor (MCR) agonists have anti-inflammatory and immunomodulatory properties mediated by receptors expressed on cells relevant to arthritis. Repository corticotropin injection (RCI; Acthar® Gel), an MCR agonist preparation, is approved as adjunctive therapy for rheumatoid arthritis (RA), but its mechanism of action in RA is unclear. This study explored the efficacy of RCI as monotherapy or adjunctive therapy with etanercept (ETN) in an established animal model of collagen-induced arthritis (CIA).

**Methods:**

After induction of CIA, rats (*n* = 10 per group) were randomized to receive subcutaneous RCI (40, 160, or 400 U/kg twice daily) alone or in combination with ETN (10 mg/kg 3 times daily), ETN alone, or vehicle (on days 13 through 19). Inflammation was assessed via changes in paw edema. Bone damage was determined by microfocal computed tomography histopathology, and immunohistochemistry. Statistical analyses were performed using a 2-way analysis of variance (ANOVA) followed by the Newman-Keuls, Dunn’s, or Dunnett’s multiple comparisons test or a 1-way ANOVA followed by the Dunnett’s or Holm-Sidak multiple comparisons test.

**Results:**

RCI administration resulted in dose-dependent decreases in ankle edema and histopathologic measures of inflammation, pannus formation, cartilage damage, bone resorption, and periosteal bone formation. RCI and ETN showed combined benefits on all parameters measured. Radiographic evidence of bone damage was significantly reduced in rats that received RCI alone or in combination with ETN. This reduction in bone density loss correlated with decreases in the number of CD68-positive macrophages and cathepsin K–positive osteoclasts within the lesions.

**Conclusions:**

As monotherapy or adjunctive therapy with ETN, RCI attenuated CIA-induced joint structural damage in rats. These data support the clinical efficacy of RCI as adjunctive therapy for patients with RA.

## Background

Rheumatoid arthritis (RA) is a chronic disease characterized by joint pain, swelling, stiffness, and reduced function. This systemic polyarthritis is associated with the inflammation of multiple joints [1]. Over long periods, the inflammation associated with RA can cause bone erosion and clinical disease that is thought to develop in response to an unidentified arthritogenic antigen [2–4]. Currently, initial approaches to RA therapy typically include disease-modifying anti-rheumatic drugs (DMARDs) such as non-steroidal anti-inflammatory drugs and methotrexate for milder disease control [5–7]. Patients who fail to respond adequately to these first-line agents or exhibit disease of rapid onset or severity are often treated with target-specific DMARDs. These include both oral small molecule agents, such as the Janus kinase inhibitor tofacitinib, and parenteral antibody or fusion protein biologics that target pro-inflammatory pathways, such as tumor necrosis factor (TNF) (eg, adalimumab, etanercept [ETN]) or interleukin-6 (eg, tocilizumab), or those that target lymphocytes such as abatacept for T cells or rituximab for B cells [8, 9].

Patients often show positive disease control after the addition of target-specific DMARDs to their treatment regimen but may lose responsiveness after several months of therapy. Exacerbations in disease activity, or flares, often necessitate a change in treatment approach with an alternative DMARD. In addition, some patients with RA fail to show adequate disease control with any of these therapeutic approaches. Thus, new or alternative therapies are needed to supplement these targeted therapeutic agents [9].

Repository corticotropin injection (RCI; Acthar® Gel) is a naturally sourced complex mixture of purified adrenocorticotropic hormone analogues and other pituitary peptides. RCI is indicated for RA as an adjunctive therapy for short-term administration to tide patients over an acute episode or exacerbation [10]. Historically, the clinical efficacy of RCI was attributed to its ability to stimulate endogenous corticosteroid production by binding to the primary receptor that mediates steroidogenesis, the melanocortin receptor (MCR) 2, in the adrenal cortex [11–13]. Recent clinical experience supports the efficacy of RCI in the treatment of RA that is resistant to either steroids or biologic DMARDS [14, 15]. Observed responses to RCI therapy in patients with treatment-refractory RA include improvements in erythrocyte sedimentation rate, C-reactive protein levels, tender and swollen joints, and pain, as well as significant improvements in Disease Activity Score in 28 joints, Health Assessment Questionnaire–Disability Index (functional disability), Functional Assessment of Chronic Illness Therapy (fatigue), and patient and physician visual analog scale scores [13, 14].

Preclinical evidence suggests that adrenocorticotropic hormone (ACTH) may modulate the immune response via binding to the known MCRs [8]. These receptors are expressed on a variety of tissues, such as immune cells (eg, B and T cells, macrophages, dendritic cells) and synovial fibroblasts [16–19], and may regulate immune functions that play a role in the pathophysiology of several diseases, including RA [20–24]. Despite its beneficial effects in patients with RA, RCI has not been previously studied in preclinical animal models of this autoimmune disease.

The purpose of the present study was to evaluate the effects of RCI when used as monotherapy or adjuvant therapy with the TNF inhibitor ETN in an established rat collagen-induced arthritis (CIA) model that exhibits joint inflammation and damage analogous to human RA and to explore potential mechanisms by which RCI may modulate the inflammatory response.

## Methods

### Model

The rat CIA model was used to characterize the effects of RCI therapy on established arthritis [25–29]. Female dark Agouti rats (weight: 120–170 g) were obtained from Harlan Laboratories, Inc. (Indianapolis, IN) and acclimated for approximately 8 days before initiation of CIA. All animal use was in accordance with the guidelines cited in the Guide for the Care and Use of Laboratory Animals [30] (Institutional Animal Care Use Committee Protocol BBP12–002). Rats were anesthetized with isoflurane, which has been shown to have a minimal effect on inflammation, and injected with 400 μL of Freund’s incomplete adjuvant (Difco, Detroit, MI) emulsified with 2 mg/mL porcine type II collagen (Chondrex, Inc., Redmond, WA) at 2 sites on the back (200 μL per site) on day 0, and then 100 μL at 1 site on day 7. Arthritis disease was represented by an increase in ankle diameter observed at day 12 and reaching a plateau at days 18 through 20.

### Protocol

Disease induction was performed on study days 0 and 7 between the time of 8:00 am and 12:00 pm, and animals were dosed in the same time frame. On study day 13 after the onset of disease, rats were assigned to treatment groups (see Additional file 1, Fig. 1 and Table 1) according to body weight and mean ankle caliper measurements. Dose selection was determined on the basis of results from an unpublished study that used a prophylactic design and showed a dose-responsive benefit. Treatments were initiated on day 13 and continued through day 19. On study day 20, all animals were anesthetized with isoflurane and were sacrificed by bleeding the descending aorta to exsanguinate (for serum collection) followed by cervical dislocation before tissue collection. Spleens were weighed, and hind paws were transected at the level of the medial and lateral malleolus for determination of paw weight as another measure of inflammation, then fixed for histopathologic evaluation and microfocal computed tomography (micro-CT).

**Fig. 1:**
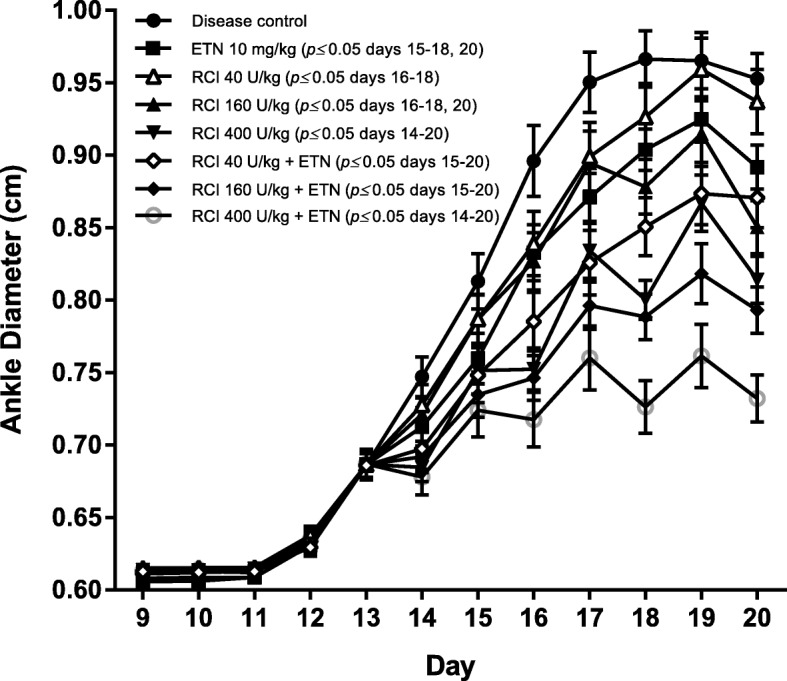
Ankle diameter measurements in rat CIA. Collagen type-II immunization of dark Agouti rats led to increased ankle diameter (mean ± SEM, *n* = 10 per group) by day 13, and these clinical parameters continued to worsen until reaching a plateau on days 18 through 20. The disease-induced increase in ankle diameter was generally dose-dependently attenuated by RCI (160 and 400 U/kg) versus the disease control group when administered after disease onset (beginning day 13). Treatment with ETN (10 mg/kg) also significantly decreased ankle diameter compared with the disease control. When administered in combination with ETN, 160 or 400 U/kg RCI further reduced ankle diameter vs ETN alone. Statistics were analyzed by 2-way ANOVA followed by a Newman-Keuls multiple comparisons test. All *p* values represent treatment compared with disease control. Abbreviations: ANOVA, analysis of variance; CIA, collagen-induced arthritis; ETN, etanercept; RCI, repository corticotropin injection; SEM, standard error of the mean

**Table 1:** Percent inhibition in disease parameters of individual and combination treatments

Disease Parameter	ETN 10 mg/kg	RCI		RCI 160 U/kg + ETN	RCI 400 U/kg + ETN
		160 U/kg	400 U/kg		
Inflammation	9%	19%^a^	26%^b^	29%^b^	55%^b^
Pannus formation	24%	17%	52%^b^	54%^b^	71%^b^
Cartilage damage	22%^a^	6%	26%^a^	44%^b^	51%^b^
Bone resorption	24%	17%	52%^b^	55%^b^	71%^b^
Periosteal bone formation	15%	12%	32%	31%	44%^c^
Paw edema AUC (day 13–20)	19%	19%	38%^b^	45%^b^	57%^b^
Serum anti-CII antibodies	2%	30%	14%	56%^d^	50%^c^

Ankles were examined microscopically by a board-certified veterinary pathologist and given a score of 0 to 5 (see Methods for additional details).

^a^ *p* ≤ 0.05 vs disease control group. ^b^ *p* ≤ 0.0001 vs disease control group. ^c^ *p* ≤ 0.01 vs disease control group. ^d^ *p* ≤ 0.001 vs disease control group. Analyzed by 2-way ANOVA followed by the Dunn’s multiple comparisons test (for inflammation, pannus formation, cartilage damage, bone resorption, and periosteal bone formation) or the Dunnett’s multiple comparisons test (for paw edema AUC and serum anti-CII antibodies)

Abbreviations: ANOVA, analysis of variance; AUC, area under the curve; CII, collagen type II; ETN, etanercept; RCI, repository corticotropin injection

Animals studies were conducted at the laboratory of Bolder BioPATH (Boulder, CO). Rat hind paws were scanned using micro-CT to visualize CIA-induced damage to joints and quantify various bone parameters at the laboratory of Numira, (Salt Lake City, UT). All animals were maintained at 5 rats/cage in plexiglass shoebox cages containing aspen wood bedding. Animals were provided Lab Diet 5001 and ad libitum tap water. Environmental controls were set to maintain temperatures of 20–26 °C with relative humidity between 30 and 70% and a 12-h light/dark cycle.

All animals were required to weigh ≥120 g prior to use in the study. Animals that showed signs of morbidity, including the loss of more than 20% body weight within 1 week, were removed from the study and humanly sacrificed via CO_2_ inhalation according to the Bolder BioPATH Program of Veterinary Care. The study protocol was reviewed and approved by the Institutional Animal Care and Use Committee of Mallinckrodt Pharmaceuticals.

### Measurements

Clinical assessment of CIA during the in-life phase was performed as previously described on days 9 through 20 [31]. Body weight and caliper measurements of right and left ankle diameters were taken on study days −1, 1, 6, and 9 through 20. Ankle caliper measurements were made with a Digitrix II micrometer (Fowler & NSK, Newton, MA). Baseline measurements were taken using 1 ankle with values rounded to 0.00254 cm. Measurements were confirmed as clinically normal (0.66–0.67 cm) by comparison with historical values for rats with a range of body weights. Experiments were performed twice with 6 animals in the naive group and 10 animals in all other groups. Animals were placed into groups such that each group consisted of similar mean ankle caliper measurements. Some animals may have been enrolled with no disease, with the assumption that disease onset would occur during the study.

### Histopathology

Ankle joints were collected and kept in 10% neutral buffered formalin for at least 24 h before placement in 5% formic acid for approximately 1 week for decalcification. When decalcification was complete, ankle joints were cut in half. Joints were processed through graded alcohols and a clearing agent, paraffin embedded, sectioned, and stained with toluidine blue to visualize cartilage proteoglycan content for general and specific evaluation of cartilage changes. Tissues from all animals were examined microscopically by a board-certified veterinary pathologist. Ankles were given scores of 0 to 5 for all histopathologic criteria. Bone resorption, ankle inflammation, and ankle cartilage damage were scored as previously described [27, 31] with the addition of 0.5, reflecting very minimal resorption affecting only marginal zones and only a few joints, minimal focal inflammation, and very minimal decrease in toluidine blue staining affecting only marginal zones, respectively. Ankle pannus was scored as previously described [32], and ankle periosteal new bone formation (measured at 16× magnification) was scored according to the following criteria: 0 = normal (no periosteal proliferation); 0.5 = minimal focal or multifocal proliferation (width measures < 127 μm [1–2 U] at any location); 1 = minimal multifocal proliferation (width at any location measures 127–252 μm [3–4 U]; 2 = mild multifocal proliferation on tarsals, diffuse in some locations, width at any location 253–441 μm [5–7 U]; 3 = moderate multifocal proliferation on tarsals, diffuse in most other locations, width at any location measures 442–630 μm [8–10 U]; 4 = marked multifocal proliferation on tarsals, diffuse at most other locations, width at any location measures 631–819 μm [11–13 U]; and 5 = severe, multifocal proliferation on tarsals, diffuse at most other locations, width at any location measures > 819 μm [> 13 U] [25, 33].

### Micro-CT analysis of joints

Hind paws were scanned with a high-resolution, volumetric micro-CT scanner (μCT40; ScanCo Medical, Zürich, Switzerland). The image data were acquired with the following parameters: 18-μm isotropic voxel resolution at 300-ms exposure time, 2000 views, and 2 frames per view scanned from the distal tibia to the mid metatarsal bones. Voxel counts were calculated using VHLab software (Numira, Salt Lake City, UT). Bone density (a measure of the amount of magnesium hydroxyapatite [mgHA] per unit volume) was calculated as the fraction of bone mineral content over total bone volume. The cortical shell is represented in the CT image by voxels of varying densities. The density of mature, solid bone voxels is > 600 mgHA/cm^3^, whereas that of young bone is < 600 mgHA/cm^3^. The numbers and percentages of voxels > 600 mgHA/cm^3^ (representing old bone, Ct_BV_high, Ct_BV_high_pc) and < 600 mgHA/cm^3^ (representing new bone, Ct_BV_low, Ct_BV_low_pc) were calculated to reveal the prevalence of new bone growth. Bone volume difference of the cortical shell (cortical volume difference), a determination of old versus new bone formation, was calculated as Ct_BV_high − Ct_BV_low.

### Immunohistochemistry

Immunohistochemistry (IHC) was performed on the Ventana Discovery-Ultra IHC/in situ hybridization automated staining platform (Ventana Medical Systems Inc., Tucson, AZ). Paraffin sections (4 μM) were used for staining with CD68 (ab125212, Abcam, Cambridge, MA) or cathepsin K (ab19027; Abcam, Cambridge, MA) antibodies. Samples were deparaffinized and treated with Ventana Cell Conditioning Solution for 32 min at 95 °C before staining. Primary antibodies were detected with anti-rabbit horse radish peroxidase and 3,3-diaminobenzidine (DAB) substrate (Ventana Roche OmniMap catalog #760–4311). Whole slide tissue sections were scanned with the NanoZoomer S210 digital slide scanner (Hamamatsu Photonics K. K., Hamamatsu, Shizuoka, Japan). Visiopharm quantitative digital pathology software (Visiopharm, Hoersholm, Denmark) was used to quantify CD68-positive or cathepsin K−positive cells using the cell classification application. The software was trained to identify DAB-positive staining, and all slides were batched and analyzed using the same algorithm. The calculated end point was the number of CD68-positive or cathepsin K−positive cells per area.

### Anti−collagen type II antibody enzyme-linked immunosorbent assay

Serum was collected at necropsy and frozen at −80^o^ C until analysis. Rat anti-collagen type II (CII) immunoglobulin G (IgG) was quantified using an enzyme-linked immunosorbent assay (ELISA) kit (Chondrex, Inc., Redmond, WA) following the manufacturer’s instructions.

### Statistical analysis

Clinical data for ankle diameter and histopathology scores were analyzed by 2-way analysis of variance (ANOVA) followed by a Newman-Keuls, Dunn’s, or Dunnet’s multiple comparison test. Bone density and cortical shell volume differences by micro-CT analysis were analyzed for differences using 1-way ANOVA followed by a Holm-Sidak multiple comparisons test using GraphPad Prism 6.0 (GraphPad Software, San Diego, CA).

## Results

### RCI suppresses inflammation and pathology in rat CIA

Rats were assigned to treatment groups according to body weight (weight: 120–170 g) and mean ankle caliper measurements (see Additional file 1, Table 1). Rats immunized with collagen developed arthritis, as assessed by paw edema by measuring ankle diameter. Treatment with ETN caused significant decreases in ankle diameter versus the disease control group (Fig. 1). Treatment with RCI also caused a significant dose-dependent reduction in ankle swelling compared with the disease control group, and the effects were additive when combined with ETN (Fig. 1). Neither ETN nor RCI treatment caused a reduction in body weight (see Additional file 1, Fig. 2). Treatment with ETN alone did not reduce paw or spleen weight (see Additional file 1, Fig. 2). At 400 U/kg, RCI significantly reduced paw and spleen weight, and paw weight was further reduced with combined RCI and ETN treatment (see Additional file 1, Fig. 2).

**Fig. 2:**
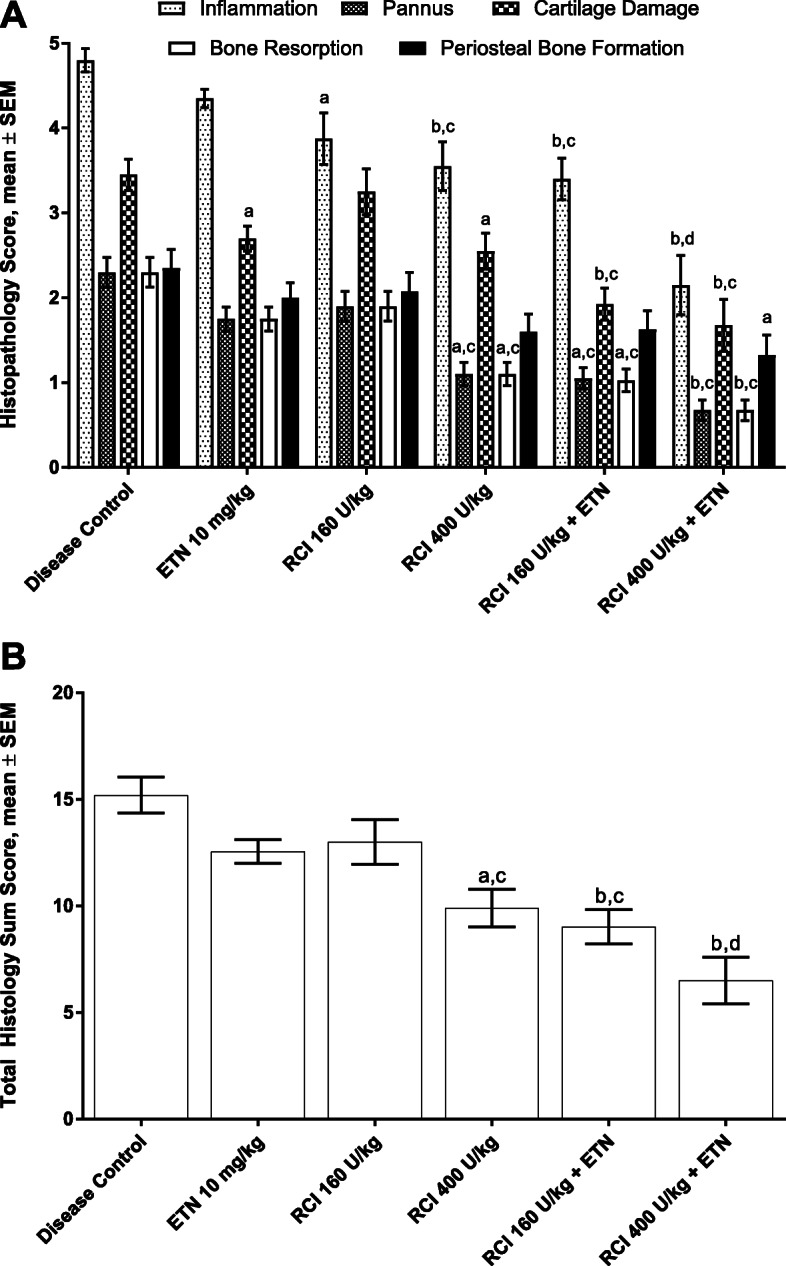
Histologic analysis of joint damage in CIA. **(a)** Disease controls showed significant increases for all individual histologic parameters of inflammation, pannus, cartilage damage, bone resorption, and periosteal bone formation (*p* ≤ 0.0001 vs naive animals). At 160 U/kg, RCI significantly inhibited ankle inflammation but had no significant beneficial effect on the other parameters. At 400 U/kg, RCI inhibited all of the histologic parameters except periosteal bone formation. Treatment with ETN alone significantly decreased cartilage damage. When ETN was combined with RCI, further decreases in most histologic parameters were observed. **(b)** Total summed histopathology score. Disease controls showed significant increases in total histopathology scores (*p* ≤ 0.0001 vs naive animals). Treatment with ETN alone and 400 U/kg RCI alone significantly decreased total histopathology scores. Greater decreases were seen when RCI and ETN were combined. ^a^ *p* ≤ 0.05 vs disease control. ^b^ *p* ≤ 0.0001 vs disease control. ^c^ *p* ≤ 0.05 vs ETN alone. ^d^ *p* ≤ 0.0001 vs ETN alone. Statistics were analyzed by 2-way ANOVA followed by a Newman-Keuls multiple comparisons test. Abbreviations: ANOVA, analysis of variance; CIA, collagen-induced arthritis; ETN, etanercept; RCI, repository corticotropin injection; SEM, standard error of the mean

Microscopic examination of diseased paws revealed a destructive polyarthritis in 100% of the disease control rats. This was characterized by synovial and periarticular edema, mixed inflammatory cell infiltration (monocyte/macrophages, neutrophils, and lymphocytes) within the joint space and synovial/periarticular tissue, marked cartilage damage, and mild to moderate bone resorption (see Additional file 1, Fig. 3; Fig. 2a and b). At 400 U/kg, RCI significantly reduced histologic evidence of injury as reflected by mean summed histopathology score and individual histologic parameters, including inflammation, pannus, cartilage damage, and bone resorption, versus the disease control group (Fig. 2a and b). Combined RCI and ETN treatment showed additional benefits on these parameters. Histologic evidence of ankle inflammation was significantly reduced in rats receiving 160 U/kg RCI compared with the disease control group, although summed histopathology scores and other parameters were not significantly attenuated at this lower dose. At 40 U/kg, RCI had no significant effects on histologic parameters (see Additional file 1, Fig. 4). Treatment with ETN alone did inhibit disease, as assessed by the summed ankle histopathology score (Fig. 2b); however, a significant effect of ETN was seen only on histologic evidence of cartilage damage compared with disease control rats (Fig. 2a).

**Fig. 3:**
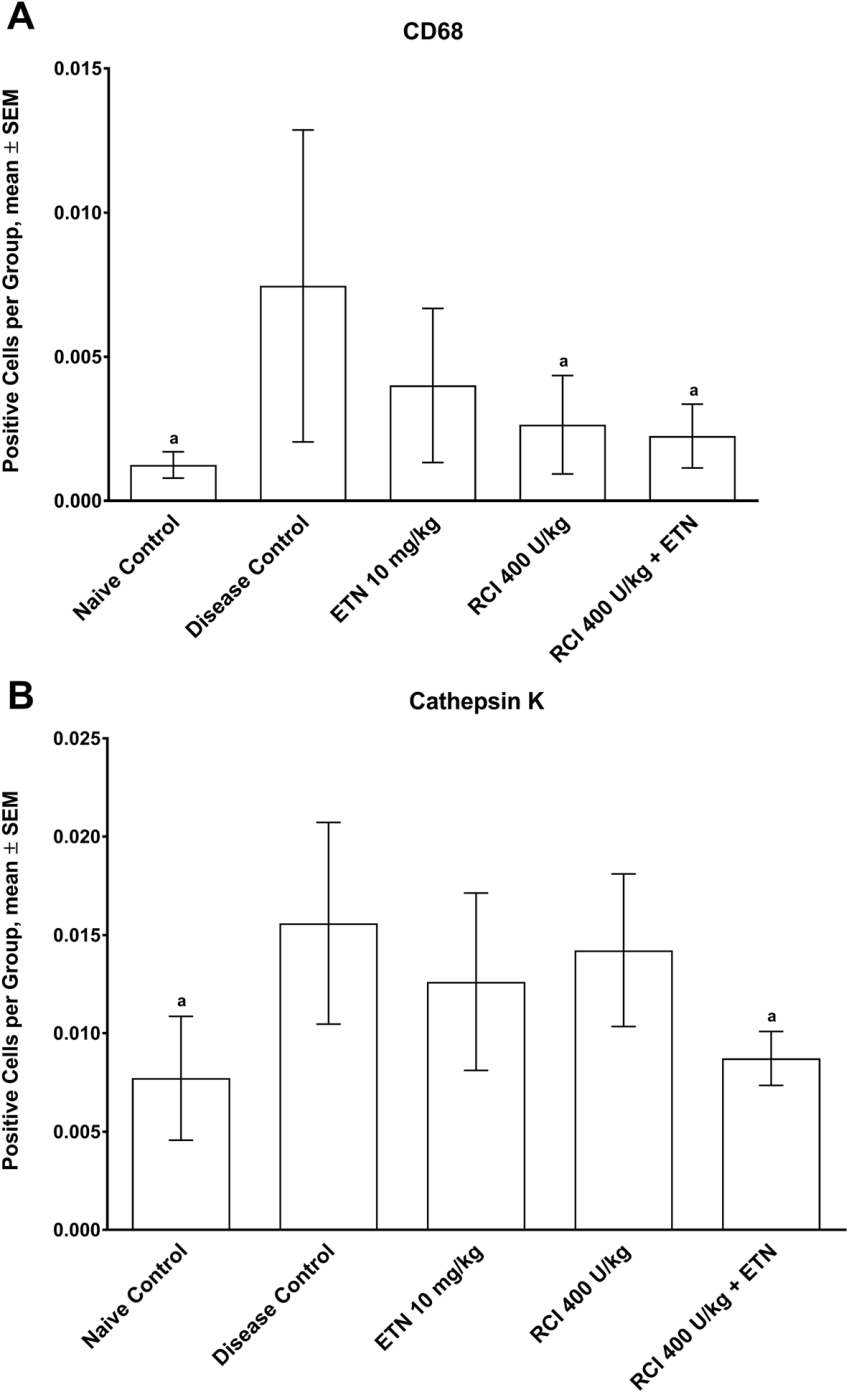
RCI treatment reduced the number of ankle monocytes/macrophages and osteoclasts in rats with CIA. Quantitative analysis scoring of **(a)** CD68–positive monocytes/macrophages and **(b)** cathepsin K–positive osteoclasts were determined using Visiopharm quantitative digital pathology software. Values are represented as mean number of positive stained cells per area ± SEM. ^a^ *p* ≤ 0.05 vs disease control. Statistics were analyzed by 1-way ANOVA followed by the Dunnett’s multiple comparisons test. Abbreviations: ANOVA, analysis of variance; CIA, collagen-induced arthritis; ETN, etanercept; RCI, repository corticotropin injection; SEM, standard error of the mean

**Fig. 4:**
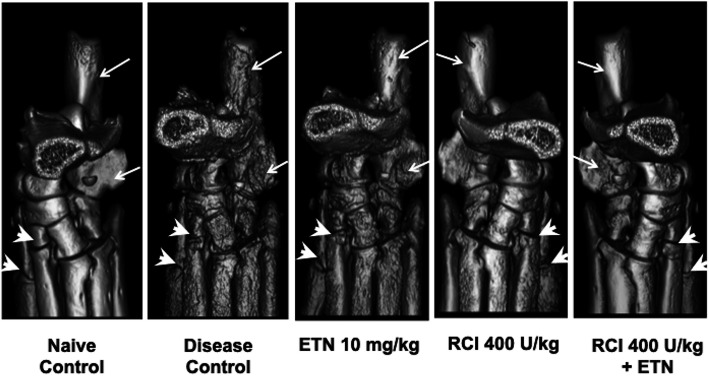
Representative micro-CT images of CIA bone destruction. Micro-CT analysis of rat ankle joints exhibited healthy bone architecture for naive animals, as shown by a smooth bone surface (arrows) and distinctive bone architecture of the ankle and foot (arrowheads). Extensive bone destruction in disease control rats is highlighted by white arrows pointing to the dimpled and roughened bone surface as well as periosteal proliferation, loss of bone mass, and fused bone joints (arrowheads). At 400 U/kg, RCI treatment decreased the amount of periosteal proliferation (arrows) as well as fused bone joints and roughened bone surface (arrowheads). When used as monotherapy, ETN 10 mg/kg was not as effective at preventing bone loss, but ETN combined with 400 U/kg RCI prevented the destruction of bone architecture, resulting in a phenotype similar to that of a naive animal. Abbreviations: CIA, collagen-induced arthritis; CT, computed tomography; ETN, etanercept; RCI, repository corticotropin injection

Table 1 shows the percent inhibition of various disease parameters after ETN and/or RCI treatment compared with the disease control group. Monotherapy with ETN showed a significant reduction in cartilage damage. However, monotherapy with RCI 400 U/kg showed significant decreases in inflammation, pannus formation, cartilage damage, bone resorption, and paw edema. These disease parameters were further reduced with combined treatment with RCI (160 or 400 U/kg) and ETN.

To better understand the cellular response in rat CIA, we used IHC analysis (see Additional file 1, Fig. 5) to semi-quantitatively determine the monocyte and osteoclast numbers as well as serum anti-CII antibody levels to evaluate the B-cell response (Fig. 3). Monocyte/macrophage infiltration and activation have been correlated with joint pain and the general inflammatory status of patients with RA [34, 35]. Ankles from rats treated with RCI or the RCI/ETN combination displayed a substantial reduction in CD68-positive macrophages versus the disease control group (Fig. 3a). Furthermore, rats treated with ETN and RCI in combination had significantly fewer cathepsin K−positive osteoclasts than did disease control rats (Fig. 3b).

B cells are believed to play a critical role in arthritis disease induction, autoantibody production, and disease progression [36]. Treatment with RCI in combination with ETN inhibited anti-CII antibody levels by > 50%. Neither ETN nor RCI alone had a significant effect on serum anti-CII antibody levels (Table 1).

### RCI inhibits structural joint damage

To further evaluate the effects of RCI alone or in combination with ETN on disease-mediated bone destruction, micro-CT of hind limb ankle joints was performed at the termination of the study. Rendering of micro-CT images demonstrated the smooth bone surface and distinctive bone architecture of the ankle and foot for naive (non-diseased) control rats. In contrast, diseased ankle joints from rats with CIA displayed extensive bone destruction, highlighted by rough bone surface and severe dimpling, periosteal proliferation, loss of bone mass, and fused bone joints. Treatment with RCI (400 U/kg) substantially improved the damaged appearance of the bone, and treatment with RCI in combination with ETN displayed even more improvement in bone appearance compared with disease control rats (Fig. 4).

To quantify changes in bone architecture, average bone density and cortical shell volume differences were calculated. As shown in Table 2, CIA disease control rats had a lower average bone density and cortical shell volume difference than naive rats. Treatment with RCI 400 U/kg inhibited bone density loss and cortical shell volume difference, reflecting a positive effect on structural damage. The protective effects of RCI on average bone density were further potentiated in combination with ETN; the CIA-induced decrease in average bone density was significantly improved by combined treatment with 400 U/kg RCI and ETN (Table 2). Furthermore, rats that received combination treatment (RCI 160 or 400 U/kg + ETN) had significantly higher cortical shell volume bone measurements than both the disease control and ETN monotherapy groups. In contrast, treatment with ETN alone had no significant effect on these bone parameters.

**Table 2:** Bone density and cortical shell volume differences

Treatment Group	Bone Density, Mean ± SD, mgHA/cm^**3**^	Mature – New Bone Volume, Mean ± SD, mm^**3**^
Naive	646.0 ± 17.8^a^	19.7 ± 1.7^b,c^
Disease control	628.5 ± 26.1	6.9 ± 7.2
ETN 10 mg/kg	605.9 ± 35.7	6.0 ± 9.9
RCI 160 U/kg	612.5 ± 22.3	8.4 ± 5.3
RCI 400 U/kg	649.0 ± 25.6^b^	13.3 ± 5.6^a,d^
RCI 160 U/kg + ETN	634.5 ± 28.4^a^	13.3 ± 5.4^a,d^
RCI 400 U/kg + ETN	655.8 ± 25.7^b,d^	15.2 ± 4.6^a,d^

^a^ *p* ≤ 0.05 vs ETN alone group; ^b^ *p* ≤ 0.0001 vs ETN alone group; ^c^ *p* ≤ 0.0001 vs disease control group; ^d^ *p* ≤ 0.05 vs disease control group

Analyzed by 1-way ANOVA followed by the Holm-Sidak multiple comparisons test.

Abbreviations: ANOVA, analysis of variance; ETN, etanercept; mgHA, magnesium hydroxyapatite; RCI, repository corticotropin injection; SD, standard deviation

## Discussion

The pathophysiology of RA encompasses a complex web of immune responses that lead to chronic joint inflammation and bone damage [1, 4]. Because MCR agonists have been shown to suppress inflammation and modulate autoimmunity [17, 18, 37–39], the present study was conducted in an animal model of RA to evaluate the efficacy of RCI as monotherapy or as adjuvant therapy with the anti-TNF agent ETN. These data demonstrate that RCI monotherapy dose-dependently inhibited both joint inflammation and bone damage in a preclinical model of established CIA. At the highest tested dose (400 U/kg), monotherapy RCI inhibited both clinical and histopathologic parameters of the CIA model to a greater degree than monotherapy 10 mg/kg ETN. Our findings are supported by and extend prior reports that suggested a role for MCR agonists in preventing or attenuating joint swelling and inflammation in animal models of immune-mediated arthritis [40–42].

The mechanism of action of RCI in reducing inflammation occurs through the activation of MCRs [43] expressed on lymphocytes, macrophages, and neutrophils [16–19], and MCR agonists have been shown to induce a number of immunomodulatory effects in these cell types [44]. These include the inhibition of cytokine production, chemotaxis, oxidative burst, adhesion molecule expression, and nuclear factor kappa-light-chain-enhancer of activated B cells (NFκB) activation [17, 18, 41, 45]. These findings suggest that RCI could suppress several biologic activities of immune cells that are known to mediate pathophysiologic effects in models of autoimmune diseases such as CIA. Antibody depletion of mature B cells has been shown to delay disease onset and autoantibody production, with significantly diminished severity of arthritis both clinically and histologically in preclinical models [46]. Blockade of TNF-α is not efficacious at decreasing humoral autoimmunity, and in fact has been reported to increase Ig levels in CIA [47, 48] and in humans with RA [49, 50]. The combination of RCI with ETN effectively decreased anti-CII Ig levels in CIA rats. Interestingly, RCI was previously reported to directly affect IgG production [51] and to inhibit circulating Ig titers [38] in disease models; however, anti-CII antibodies in this study were not significantly decreased by RCI alone.

The CIA model is associated with bone destruction. In addition to attenuating joint inflammation and paw swelling, RCI treatment dose-dependently reduced CIA-induced effects on cartilage damage and bone resorption as assessed by histologic examination. Micro-CT analysis supported positive effects of RCI on bone remodeling; significant improvement in the cortical bone volume difference and a trend for treatment-related improvement of mean bone density were observed. The beneficial effects of RCI have also been observed in patients with persistently active RA despite the use of corticosteroid/DMARD therapy, where bone turnover markers remained stable with RCI treatment, suggesting a minimal impact on bone metabolism [52].

Other MCR agonists have also been shown to be beneficial in animal models of arthritis [22, 42, 53, 54]. Intra-arterial administration of ACTH_1–39_ inhibits inflammation in a corticosterone-independent manner in gouty arthritis, likely through interactions with MCRs [22]. Reduced bone remodeling in RCI-treated animals may be secondary to the effect of RCI on inhibiting joint inflammation; however, MCR expression has been described on osteoclasts, osteoblasts, and chondrocytes [55, 56], and prior evidence suggests that melanocortin ligands may directly impact cartilage and bone physiology [42]. For example, ACTH and α melanocyte-stimulating hormone (αMSH) have been shown to increase the proliferation of chondrocyte progenitors and induce differentiation of mature chondrocytes [55, 57] in addition to enhancing matrix production by increasing collagen production and decreasing matrix metalloproteinase levels [55, 58–60]. αMSH inhibits the release of inflammatory cytokines from chondrocytes and has chondro-protective activity [59]. Furthermore, ACTH and αMSH have been shown to increase the survival and maturation of osteoblasts as well as regulate osteoclast numbers [57, 61]. Osteoclasts are highly specialized cells of hematopoietic lineage that are uniquely responsible for bone resorption.

To further explore the potential mechanism underlying the bone-protective effects of RCI and combination therapy, IHC staining for osteoclasts was performed. Compared with the naive group, the disease control group had more CD68-positive macrophages and cathepsin K−positive osteoclasts (Fig. 3). Treatment with RCI and ETN alone substantially diminished the number of CD68-positive macrophages in inflamed joints. Furthermore, the number of cathepsin K−positive cells was dramatically reduced after combination treatment. MCR-deficient mice have displayed an up-regulation of *Rankl* in their joints, with an increase in bone erosion and mature osteoclasts [38], which suggests that MCRs could modulate mediators that effect osteoclast number, differentiation, and function.

Limitations of this study may include lack of an RCI dosing regimen that would provide maximum clinical benefit, which has not been fully elucidated. RCI is a naturally sourced complex mixture of ACTH analogs and other pituitary peptides, so there could be multiple mechanisms of action that contribute to the response we see here. For example, the effects of RCI on reduced bone remodeling may be secondary as a result of anti-inflammatory properties. In addition, this study did not compare the effects of RCI with more conventional standard of care therapies, such as corticosteroids, which would be an important assessment for future research.

## Conclusions

In summary, this is the first study to illustrate the benefits of RCI for inflammation and bone remodeling in established rat CIA, a preclinical model that exhibits joint inflammation and damage analogous to human RA [25]. When administered as adjunctive therapy with ETN, RCI additively reduced disease manifestations, including ankle edema and histopathologic measures of inflammation, pannus formation, cartilage damage, bone resorption, and periosteal bone formation. These preclinical data demonstrate a pivotal role for RCI in controlling inflammation and bone damage in immune-mediated arthritis and lend support to the efficacy of RCI in the treatment of patients with active RA.

## Supplementary information

**Additional file 1: Table S1.** Experimental conditions. **Fig. S1.** Study design. **Fig. S2.** Analysis of body, spleen, and paw weight. **Fig. S3.** Histopathology images of the ankle. **Fig. S4.** Histologic analysis of joint damage in CIA. **Fig. S5.** IHC images displaying CD68-positive macrophages and cathepsin K-positive osteoclasts.

## Data Availability

The datasets used and/or analyzed during the current study are available from the corresponding author on reasonable request

## Abbreviations

RA: rheumatoid arthritis
DMARDs: disease-modifying anti-rheumatic drugs
TNF: tumor necrosis factor
ETN: etanercept
RCI: repository corticotropin injection
MCR: melanocortin receptor
ACTH: adrenocorticotropic hormone
CIA: collagen-induced arthritis
micro-CT: microfocal computed tomography
mgHA: magnesium hydroxyapatite
IHC: immunohistochemistry
DAB: 3,3-diaminobenzidine
CII: collagen type II
IgG: immunoglobulin G
ELISA: enzyme-linked immunosorbent assay
ANOVA: analysis of variance
NFκB: nuclear factor kappa-light-chain-enhancer of activated B cells
αMSH: alpha melanocyte-stimulating hormone
