# Supplementary material for: Repository corticotropin injection attenuates collagen-induced arthritic joint structural damage and has enhanced effects in combination with etanercept
Source: BMC Musculoskelet Disord. 2020 Aug 31;21:586. doi: 10.1186/s12891-020-03609-3 (PMC7460755; doi:10.1186/s12891-020-03609-3)
Supplement: Supplementary file 1 — Additional file 1: Table S1. Experimental conditions. Fig. S1. Study design. Fig. S2. Analysis of body, spleen, and paw weight. Fig. S3. Histopathology images of the ankle. Fig. S4. Histologic analysis of joint damage in CIA. Fig. S5. IHC images displaying CD68-positive macrophages and cathepsin K-positive osteoclasts. [file 12891_2020_3609_MOESM1_ESM.docx]

| **Additional Table 1.** Experimental conditions | | | |
| --- | --- | --- | --- |
| **Group** | **n** | **CIA?** | **Treatment** |
| Naive control | 6 | No | Placebo gel^a^: 5 mL/kg SC Q2D  Sterile water^b^: 1 mL/kg SC Q3D |
| Disease control | 10 | Yes | Placebo gel^a^: 5 mL/kg SC Q2D  Sterile water^b^: 1 mL/kg SC Q3D |
| RCI 40 U/kg | 10 | Yes | RCI: 40 U/kg SC Q2D |
| RCI 160 U/kg | 10 | Yes | RCI: 160 U/kg SC Q2D |
| RCI 400 U/kg | 10 | Yes | RCI: 400 U/kg SC Q2D |
| ETN | 10 | Yes | ETN: 10 mg/kg SC Q3D |
| RCI 40 U/kg + ETN | 10 | Yes | RCI: 40 U/kg SC Q2D  ETN: 10 mg/kg SC Q3D |
| RCI 160 U/kg + ETN | 10 | Yes | RCI: 160 U/kg SC Q2D  ETN: 10 mg/kg SC Q3D |
| RCI 400 U/kg + ETN | 10 | Yes | RCI: 400 U/kg SC Q2D  ETN: 10 mg/kg SC Q3D |
| ^a^ Vehicle for RCI. ^b^ Vehicle for ETN.  Rats were SC injected behind the head, between the shoulder blades.  Abbreviations: CIA, collagen-induced arthritis; ETN, etanercept; Q2D, every 2 days; Q3D, every 3 days; RCI, repository corticotropin injection; SC, subcutaneously. | | | |

**

**

**Additional Figure 1** Study design. Animals were acclimated for approximately 8 days prior to the induction of CIA, where collagen was injected on days 0 and 7. Ankle caliper and body weight measurements were taken prior to treatment with placebo gel, sterile water, RCI, or ETN. Treatments were initiated on day 13, as detailed in Additional Table 1, and continued through day 19. Ankle caliper, body weight, and clinical scoring of CIA were assessed on days 9 through 20.

Abbreviations: CIA, collagen-induced arthritis; ETN, etanercept; Q2D, every 2 days; Q3D, every 3 days; RCI, repository corticotropin injection.

**

**

**Additional Figure 2** Analysis of body (A), spleen (B), and paw (C) weight. Statistics were analyzed by a 1-way ANOVA followed by the Holm-Sidak multiple comparisons test.

^a^ *p*≤0.05 vs disease control. ^b^ *p*≤0.0001 vs disease control. ^c^ *p*≤0.05 vs ETN alone. ^d^ *p*≤0.0001 vs ETN

Abbreviations: ANOVA, analysis of variance; ETN, etanercept; RCI, repository corticotropin injection; SEM, standard error of the mean.

**
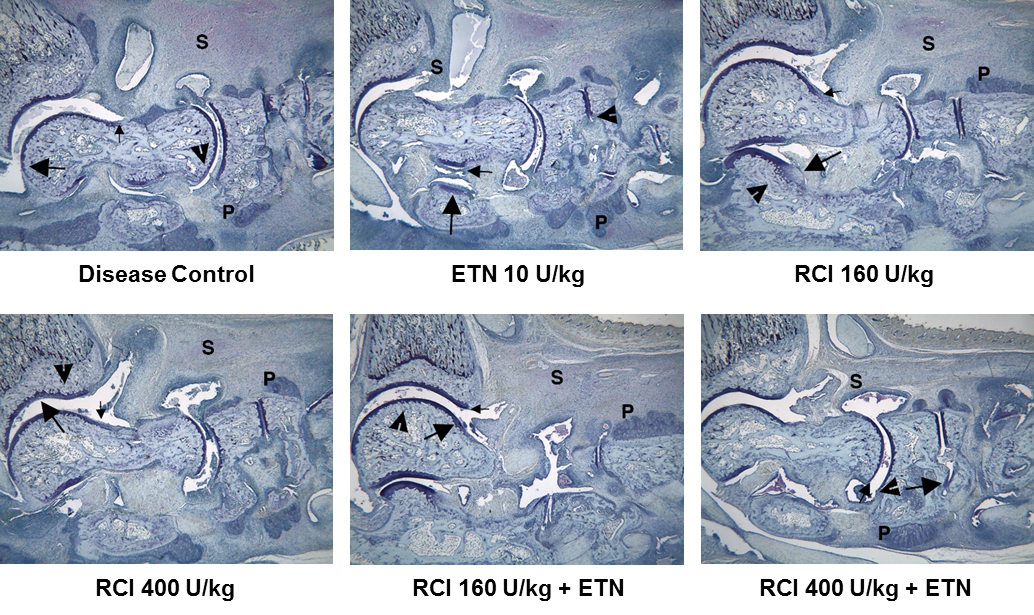
**

**Additional Figure 3** Histopathology images of the ankle. S indicates severe inflammation, the large arrow indicates marked cartilage damage, the small arrow indicates mild pannus, the arrowhead indicates bone resorption, and P indicates periosteal bone formation. Rat Collagen Methods 5-12-20

Inflammation was scored as follows: 0=normal (approximate paw thickness=80-90 U on 16X, 5040-5733 µm); 0.5=minimal, generally focal or multifocal inflammation (approximate paw thickness=80-90 U on 16X, 5040-5733 µm); 1=minimal infiltration of inflammatory cells in synovium/periarticular tissue (approximate paw thickness=90-100 U on 16X, 5670-6300 µm); 2=mild infiltration (approximate paw thickness=91-110 U on 16X, 5733-6930 µm); 3=moderate infiltration with moderate edema (approximate paw thickness=111-120 U on 16X, 6993-7560 µm); 4=marked infiltration with marked edema (approximate paw thickness=121-130 U on 16X, 7523-8190 µm); 5=severe infiltration with severe edema (approximate paw thickness=131-140 U on 16X, 8253-8820 µm); 6=very severe infiltration with very severe edema (approximate paw thickness=>140 U on 16X, >8820 µm).

Cartilage damage was scored as follows: 0=normal; 0.5=minimal decrease in toluidine blue staining, affects only marginal zones and only a few joints, less than 1% total cartilage loss; 1=minimal to mild loss of toluidine blue staining with minor chondrocyte loss and collagen disruption, 1%-10% total loss; 2=mild loss of toluidine blue staining with focal mild (superficial) chondrocyte loss and/or collagen disruption, 11%-25% total loss; 3=moderate loss of toluidine blue staining with multifocal moderate (depth to middle zone) chondrocyte loss and/or collagen disruption, smaller tarsals affected to 1/2-3/4 depth with rare areas of full thickness loss, 26%-50% loss; 4=marked loss of toluidine blue staining with multifocal marked (depth to deep zone) chondrocyte loss and/or collagen disruption, 1 or 2 small tarsals surfaces have full thickness loss of cartilage, 51%-75% total loss; 5=severe diffuse loss of toluidine blue staining with multifocal severe (depth to tide mark) chondrocyte loss and/or collagen disruption affecting more than 2 cartilage surfaces, greater than 75% total loss.

Pannus was scored as follows: 0=normal; 0.5=minimal infiltration of pannus in cartilage and subchondral bone, affects only marginal zones and affects only a few joints, less than 1% of area at risk affected; 1=minimal infiltration of pannus in cartilage and subchondral bone, primarily affects marginal zones, 1%-10% of area at risk affected; 2=mild infiltration (<1/4 of tibia or tarsals at marginal zones), 11%-25% of area at risk affected; 3=moderate infiltration (1/4 to 1/3 of tibia or small tarsals affected at marginal zones), 26%-50% of area at risk affected; 4=marked infiltration (1/2-3/4 of tibia or tarsals affected at marginal zones), 51%-75% of area at risk affected; 5=severe infiltration (>3/4 of tibia or tarsals affected at marginal zones, severe distortion of overall architecture), greater than 75% of area at risk affected.

Bone resorption was scored as follows: 0=normal; 0.5=minimal resorption, affects only marginal zones and only a few joints, less than 1% of area at risk affected; 1=small areas of resorption at distal tibia and marginal zones of tarsals, apparent on low magnification, generally low numbers of osteoclasts, 1%-10% of area at risk affected; 2=more numerous areas of resorption but no full thickness defects in the distal tibia and mild loss of medullary bone across the growth plate, readily apparent on low magnification, more numerous osteoclasts, mainly marginal zones of tarsals affected, 11%-25% of area at risk affected; 3=obvious resorption of tibial medullary trabecular and cortical bone without full thickness defects in both tibial cortices, incomplete loss of medullary bone across the growth plate, numerous osteoclasts, may have a few small areas of resorption in tarsals especially at marginal zones, or may have more tarsal (especially calcaneus) resorption and less tibial damage, 26%-50% of area at risk affected; 4=full thickness defects in distal tibial cortical bone (one or both cortices), often with distortion of profile of the remaining cortical surface, marked loss of medullary bone across the growth plate, numerous osteoclasts, small areas of resorption may be present in a few tarsals but generally tarsal involvement is minimal to mild, 51%-75% of area at risk affected; 5=full thickness defects in distal tibial cortical bone (both cortices), often with distortion of profile of remaining cortical surface, severe loss of medullary bone across the growth plate, numerous osteoclasts, larger areas of resorption present in several tarsal bones (especially calcaneus), greater than 75% of area at risk affected.

Periosteal new bone formation was scored as follows: 0=normal, no periosteal proliferation; 0.5=minimal focal or multifocal proliferation, measures less than 127 µm width (1-2 U at 16X) at any location; 1=minimal multifocal proliferation, width at any location measures 127 µm-252 µm (3-4 U at 16X); 2=mild multifocal on tarsals, diffuse in some locations, width at any location 253 µm-441 µm (5-7 U at 16X); 3=moderate multifocal on tarsals, diffuse in most other locations, width at any location measures 442 µm-630 µm (8-10 U at 16X); 4=marked multifocal on tarsals, diffuse at most other locations, width at any location measures 630 µm-819 µm (11-13 U at 16X); 5=severe, multifocal on tarsals, diffuse at most other locations, width at any location measures >819 µm (>13 U at 16X).

Abbreviations: ETN, etanercept; IHC, immunohistochemistry; RCI, repository corticotropin injection; U, units.

**
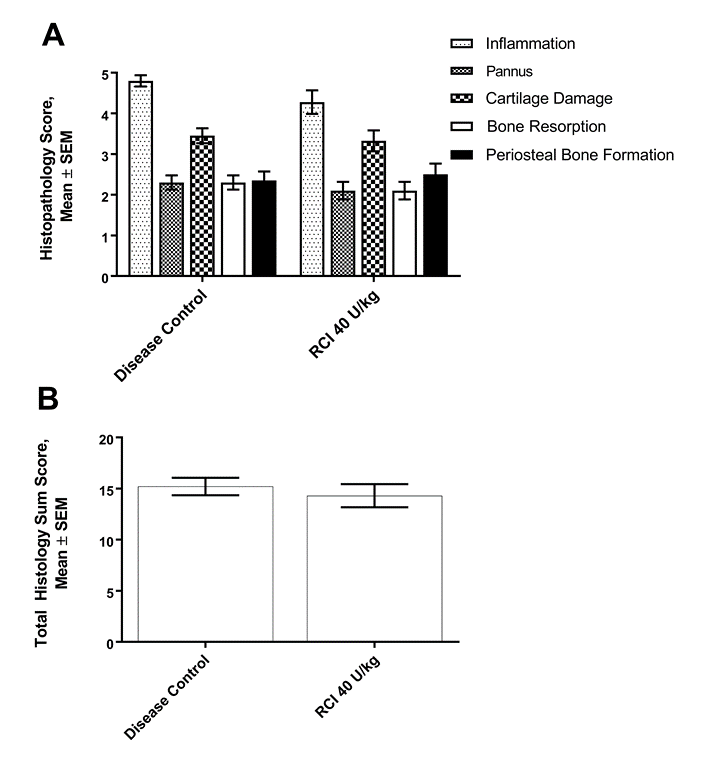
**

**Additional Figure 4** Histologic analysis of joint damage in CIA. **(A-B)** At 40 U/kg, RCI did not have a significant effect on histological parameters compared to the disease control.

Statistics were analyzed by 2-way ANOVA followed by the Newman-Keuls multiple comparisons test.

Abbreviations: ANOVA, analysis of variance; CIA, collagen-induced arthritis; RCI, repository corticotropin injection; SEM, standard error of the mean.

**
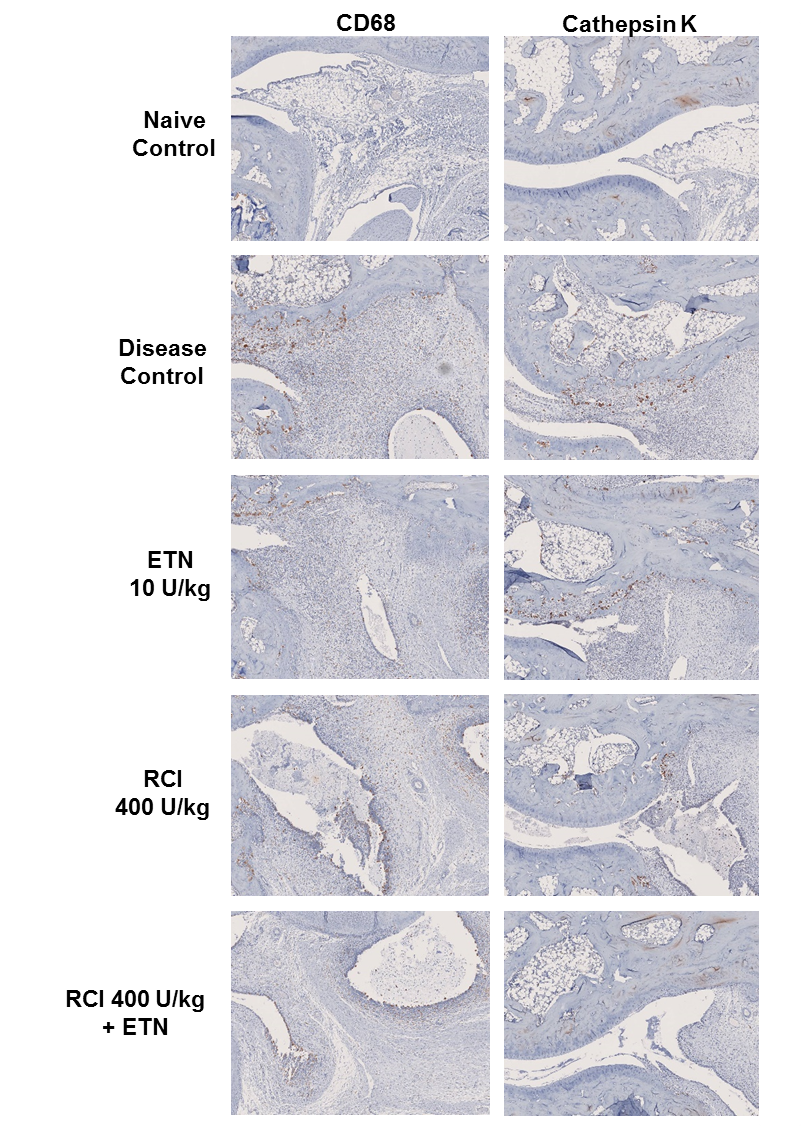
**

**Additional Figure 5** IHC images displaying CD68-positive macrophages and cathepsin K-positive osteoclasts.

Abbreviations: ETN, etanercept; IHC, immunohistochemistry; RCI, repository corticotropin injection.
